# Transcriptome-Based Evaluation of Optimal Reference Genes for Quantitative Real-Time PCR in Yak Stomach throughout the Growth Cycle

**DOI:** 10.3390/ani13050925

**Published:** 2023-03-03

**Authors:** Qi Min, Lu Yang, Yu Wang, Yili Liu, Mingfeng Jiang

**Affiliations:** 1 Institute of Qinghai-Tibetan Plateau, Southwest Minzu University, Chengdu 610041, China; 2 College of Animal and Veterinary Sciences, Southwest Minzu University, Chengdu 610041, China

**Keywords:** yak, stomach, transcriptome-wide, reference gene, RT-qPCR

## Abstract

**Simple Summary:**

The stomach is one of the primary sites for the digestion and absorption of nutrients. Quantifying related gene expression patterns using quantitative real-time PCR (RT-qPCR) is conducive to further understanding the molecular mechanisms underlying nutrition metabolism in the yak stomach. The authenticity of RT-qPCR data is affected by the selection of reference genes. Unfortunately, no studies have demonstrated suitable reference genes for the normalization of RT-qPCR data in the yak stomach. In this study, 15 candidate reference genes (CRGs) were identified according to transcriptome sequencing (RNA-seq) results and the previous literature. Five algorithms were used to evaluate the stability of the CRGs across the entire developmental stage in the yak stomach. *RPS15*, *MRPL39*, and *RPS23* were found to be the most stable genes in the yak stomach from birth to adulthood. This study indicates the appropriate reference genes for gene expression analysis via RT-qPCR in the yak stomach.

**Abstract:**

Efficient nutritional assimilation and energy metabolism in the stomachs of yaks contribute to their adaption to harsh environments. Accurate gene expression profile analysis will help further reveal the molecular mechanism of nutrient and energy metabolism in the yak stomach. RT-qPCR is regarded as an accurate and dependable method for analyzing gene expression. The selection of reference genes is essential to obtain meaningful RT-qPCR results, especially in longitudinal gene expression studies of tissues and organs. Our objective was to select and validate optimal reference genes from across the transcriptome as internal controls for longitudinal gene expression studies in the yak stomach. In this study, 15 candidate reference genes (CRGs) were determined according to transcriptome sequencing (RNA-seq) results and the previous literature. The expression levels of these 15 CRGs were quantified using RT-qPCR in the yak stomach, including the rumen, reticulum, omasum and abomasum at five stages: 0 days, 20 days, 60 days, 15 months and three years old (adult). Subsequently, the expression stabilities of these 15 CRGs were evaluated via four algorithms: geNorm, NormFinder, BestKeeper and the comparative C_T_ method. Furthermore, RefFinder was employed to obtain a comprehensive ranking of the stability of CRGs. The analysis results indicate that *RPS15*, *MRPL39* and *RPS23* are the most stable genes in the yak stomach throughout the growth cycle. In addition, to verify the reliability of the selected CRGs, the relative expression levels of *HMGCS2* were quantified via RT-qPCR using the three most stable or the three least stable CRGs. Overall, we recommend combining *RPS15*, *MRPL39* and *RPS23* as reference genes for the normalization of RT-qPCR data in the yak stomach throughout the growth cycle.

## 1. Introduction

The yak (*Bos grunniens*), a precious domesticated ruminant, also known as the “boat on the plateau”, is mostly found on the Qinghai-Tibetan Plateau and nearby areas at an altitude above 3000 m. As the most significant livestock in this region, yaks are capable of surviving and providing milk, meat, hair and cheese for local herders in a hostile environment [1]. A previous study found that efficient nutritional assimilation and energy metabolism in the yak stomach contributes to their adaption to a harsh environment [2]. In ruminants, the stomach and small intestine are mostly where nutrients are digested and absorbed [3]. Additionally, the development of the yak stomach at different stages plays a vital role in digestive ability and nutrient supply [4]. Thus, accurate analysis of gene expression profiles in the yak stomach are of major priority to further reveal the molecular mechanisms of nutrient and energy metabolism.

As a typical ruminant, a remarkable feature of the yak is that it has a complex stomach consisting of four gastric compartments: rumen, reticulum, omasum and abomasum [5]. The first three compartments of the compound stomach (i.e., rumen, reticulum and omasum) are commonly referred to as the “forestomach” and perform cooperative functions [6]. They serve as fermentative chambers where bacteria break down the ingested cellulose, producing enormous amounts of gas [7]. By contrast, only the abomasum can generate digestive juices and gastric enzymes [7]. Hence, the abomasum is also called the true stomach. In newborn ruminants, dietary requirements are fulfilled by the uptake of colostrum, which is digested in the abomasum to provide energy and essential nutrients, as well as immunity molecules [8]. In comparison, the rumen acts as the primary location of digestion and absorption in grown ruminants, and microorganisms decompose ingested feed in the rumen to produce volatile fatty acids (VFA) that serve as the main source of energy [9]. Understanding which genes in the stomach are crucial for nutrient absorption and digestion and how they might be regulated to contribute to growth and maintenance is a major concern in the field of yak research.

RT-qPCR is extensively used for the analysis of gene expression patterns due to its sensitivity, accuracy and specificity, as well as practical simplicity [10,11]. However, several drawbacks such as nucleic acid quality, poor choice of primers or probes and inappropriate data and statistical analyses encumber the authenticity of RT-qPCR results [12]. Therefore, various strategies have been applied to normalize RT-qPCR results. The use of reference genes that are not affected by study conditions is a generally accepted strategy for normalizing RT-qPCR data [13]. Despite the fact that several genes such as *ACTB* and *GAPDH* are commonly employed as reference genes in a wide range of studies, it is unlikely that any genes have enough overall expression stability to be appropriate for any kind of experiment [14]. Therefore, it is essential to select reliable reference genes for the specific experimental context under study. To date, no studies have shown suitable reference genes for the normalization of RT-qPCR data in the yak stomach.

Many studies have concentrated on verifying subsets of frequently used reference genes for specific experimental contexts [14]. However, it is biased to select the CRGs from a minority of genes and assume that at least a few of those genes are appropriate for the certain experimental context. The emergence of high-throughput RNA-seq technology provides a novel strategy for identifying reference genes [15]. Based on the RNA-seq dataset, CRGs with stable expression and high abundance were preliminarily selected. Subsequently, the expression levels of CRGs were quantified using RT-qPCR and their stabilities were evaluated using geNorm [16], NormFinder [17], BestKeeper [18] and the comparative C_T_ method [19]. This strategy has been successful for identifying reference genes for fish [20], Holstein cows [21], goats [22], and so on.

The purpose of this study was to select and validate reliable reference genes from across the transcriptome that can serve as internal controls for longitudinal gene expression studies in the yak stomach throughout the growth cycle.

## 2. Materials and Methods

### 2.1. Animals and Sample Collection

All the experimental protocols were approved by the Institutional Animal Care and Use Committee of Southwest Minzu University (permit number: 2020-07-02-11). All Maiwa yaks were raised in Hongyuan County of Sichuan Province and fed with natural lactation and pasture. A total of 15 Maiwa yaks (7 males and 8 females) were selected from the same herd at 5 different growth stages: 0 days (lactating stage), 20 days (lactating stage and starting to graze), 60 days (lactating stage and graze stage), 15 months (graze stage but still lactating) and 3 years old (natural graze stage). For sample collection, three separate yaks of each age were slaughtered. The stomach tissues of the yaks, including rumen, reticulum, omasum and abomasum, were rinsed immediately in 0.1% DEPC water after slaughter and frozen in liquid nitrogen until processing for total RNA extraction.

### 2.2. RNA Extraction and cDNA Synthesis

The total RNA of the rumen, reticulum, omasum and abomasum tissues were extracted using the mirVana miRNA Isolation Kit (Invitrogen, Carlsbad, CA, USA) following the manufacturer’s protocol. The purity and concentration of total RNA were confirmed using the NanoDrop2000 spectrophotometer (Thermo Fisher Scientific, Waltham, MA, USA). The integrity of total RNA was assessed using 1% agarose gel electrophoresis. The cDNA was generated from 1000 ng total RNA using the PrimeScript RT reagent Kit with gDNA Eraser (TaKaRa, Dalian, China) in a reaction mixture of 20 µL. The cDNA was stored at −80 °C until required.

### 2.3. Selection of CRGs

Based on our previous RNA-seq results of the compound stomach at five stages in fifteen yaks (unpublished data), 7 CRGs, ribosomal protein S15 (*RPS15*), ribosomal protein S23 (*RPS23*), 3-monooxygenase/tryptophan 5-monooxygenase activation protein zeta (*YWHAZ*), ribosomal protein L13a (*RPL13A*), β-actin (*ACTB*), ribosomal protein S9 (*RPS9*) and glyceraldehyde-3-phos-phate dehydrogenase (*GAPDH*), were selected according to the fragments per kilobase of exon model per million mapped reads (FPKM) and the coefficient of variation (CV, %). The value of FPKM was higher than 100 and the CV value was less than 20%. FPKM = cDNA fragments/[mapped fragments (millions) × transcript length (kb)] and CV = standard deviation (SD) _FPKM_/Mean_FPKM_ × 100%. Subsequently, based on the previous literature, eight genes were selected as CRGs: ubiquitously expressed prefoldin-like chaperone (*UXT*), dystrobrevin binding protein (*DBNDD2*), DEAD box polypeptide 54 (*DDX54*), hydroxymethylbilane synthase (*HMBS*), protein phosphatase 1 regulatory inhibitor subunit 11 (*PPP1R11*), mitochondrial ribosomal protein S15 (*MRPS15*), mitochondrial ribosomal protein L39 (*MRPL39*) and TATA box binding protein (*TBP*).

### 2.4. Primer Pairs Design

Primers for RT-qPCR were designed using Primer-BlAST with a length of 20 ± 3 bases and amplicon sizes ranging from 100 to 150 bp. The sequences of the CRGs were obtained from NCBI (https://www.ncbi.nlm.nih.gov/ accessed on 25 June 2022). The primer specificity of each CRG was verified using 2% agarose gel electrophoresis and melting curve analysis. To validate the specificity of each primer pair, the products of PCR were purified and sequenced using a 3730 DNA analyzer (ABI, Carlsbad, CA, USA), and the sequencing results were compared with all potential transcript sequences in NCBI using BLAST.

### 2.5. RT-qPCR Assay

All RT-qPCR assays were carried out in triplicate for each sample using the LightCycler 96 System (Roche Diagnostics, Indianapolis, IN, USA). The total volume of each reaction mixture was 20 µL, including 10 µL of TB Green Premix Ex Taq II (TaKaRa, Dalian, China), 2 µL of diluted cDNA, 0.5 µL of each of 10 μM forward and reverse primers and 7 µL of RNase Free dH_2_O. The PCR program was conducted as follows: 95 °C for 30 s (pre-denaturation), 40 cycles of 95 °C for 5 s and 60 °C for 30 s (quantitative analysis), 95 °C for 5 s and 60 °C for 1 min (melting curves analysis). To determine the correlation coefficient (R^2^) and amplification efficiency (E) for each primer pair, a five-point standard curve was generated using a five-fold dilution of cDNA. The correlation coefficient (R^2^) and amplification efficiency (E) of each primer pair were calculated using the LightCycler 96 System. A modified Pfaffl equation was used to determine the relative quantity (RQ) of each gene [23]:

$${\mathrm{RQ}}_{\mathrm{sample}}={\;E\;}^{\left(\mathrm{Cq}\;\left(\mathrm{Calibrator}\right)-\;\mathrm{Cq}\;\left(\mathrm{Sample}\right)\;\right)}$$

C_q_ (calibrator) = C_q_ for the arithmetic mean of all samples at 5 stages, C_q_ (sample) = C_q_ for the sample. The formula for calculating the relative expression level of a target gene is as follows:

$$\mathrm{Relative}\;\mathrm{gene}\;\mathrm{expression}=\frac{{\mathrm{RQ}}_{\mathrm{GOI}}}{\mathrm{Geomean}\left[{\mathrm{RQ}}_{\mathrm{REFs}}\right]}$$

RQ_GOI_: the RQ value of the target gene, Geomean[RQ_REFS_]: the geometric mean of the RQ value of selected reference genes. The normalization factor (NF) was calculated using the geometric mean of the RQ value of the selected reference genes [23].

### 2.6. Stability Analysis of CRGs

The expression stability of 15 CRGs was evaluated using 4 algorithms: geNorm, NormFinder, BestKeeper and the comparative C_T_ method. In addition, RefFinder (http://blooge.cn/RefFinder/ accessed on 10 October 2022) was used to synthesize the evaluation results of the above four algorithms to give an overall ranking.

### 2.7. Validation of Optimal Reference Gene Combinations

*HMGCS2* is the key rate-limiting enzyme in the ketogenic pathway and plays an important role in the digestion and absorption of nutrients in the stomach. The expression levels of *HMGCS2* were quantified using RT-qPCR to validate the selected reference genes. The expression levels of *HMGCS2* in the stomach at 5 stages were normalized using the three most stable gene combinations and the three most unstable gene combinations identified from this study. The relative mRNA expression of *HMGCS2* was calculated using the 2^−∆∆Ct^ method. In addition, statistical significance was analyzed using one-way analysis of variance via SPSS 25.0 software (IBM, Armonk, NY, USA). A *p* value below 0.05 was regarded as statistically significant.

## 3. Results

### 3.1. Quality Control of Total RNA

The 260/280 ratio of total RNA for each sample ranged from 1.8 to 2.2, and the purity and concentration were qualified for subsequent experiments (Table S1). The RNA of all samples clearly displayed two prospective bands at 18 s and 28 s without any signs that the products were degraded (Figure S1). The above results indicate that the RNAs of all samples were equipped for cDNA synthesis.

### 3.2. Selection of CRGs Based on RNA-seq Data and Previous Literature

The criteria for preliminary selection of reference genes were relatively high transcriptome abundance and low expression variation [15]. As a result, preliminary selection comprised genes with relatively high transcriptome abundance (FPKM > 100) as identified by the mean FPKM value and low variability as identified by the coefficient of variation (CV < 20%). A total of 80 CRGs were preliminarily selected using our previous RNA-seq results of the stomach at five stages in fifteen yaks (Table S2). Furthermore, 7 genes (*RPS15*, *RPS23*, *YWHAZ*, *RPL13A*, *ACTB*, *GAPDH*, and *RPS9*) were considered as CRGs due to their lower CV values, higher FPKM values, and easier primer designs. In addition, eight CRGs were selected based on previous studies. Among these CRGs: *UXT*, *HMBS*, *MRPS15*, *PPP1R11*, *MRPL39* and *TBP* were validated to be appropriate reference genes for RT-qPCR in yak [13,24,25,26]. Additionally, *DBNDD2* and *DDX54* were verified as suitable reference genes for RT-qPCR in the rumen epithelium of cows [27]. In conclusion, 15 genes were chosen as CRGs for further evaluation.

### 3.3. Characteristics of Primer Pairs

The details of primer pairs of 15 CRGs are displayed in Table 1. Primer pairs of amplification efficiency (%) ranged from 91 to 109%, the amplicon’s size lay in 100–286 bp and the R^2^ of each primer pair was not less than 0.99. The specificity of primer pairs for each gene was verified via 2% agarose gel electrophoresis (Figure S2) and melting curve analysis (Figure S3). To further validate the specificity of each primer pair, the products of PCR were purified and sequenced. Then, the sequencing results were compared with all potential transcript sequences in NCBI using BLAST (Table S3).

### 3.4. RT-qPCR Analysis for CRGs

**Figure 1:**
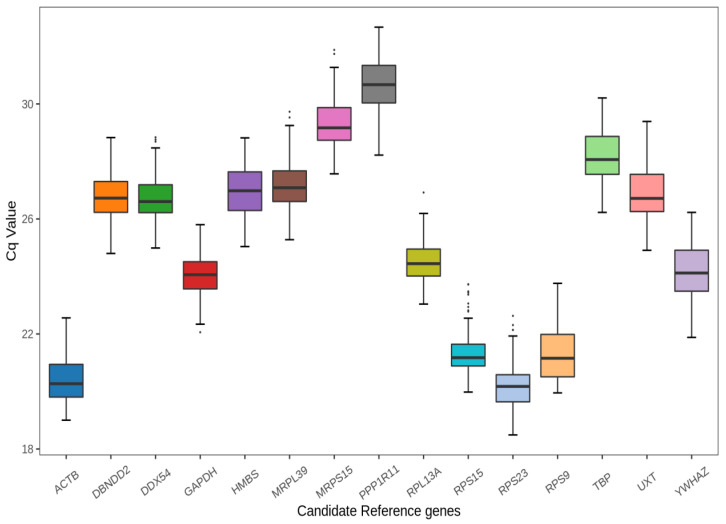
The mean C_q_ values of 15 CRGs in a total of 60 samples of yak stomach including rumen, reticulum, omasum and abomasum at 5 developmental stages. The C_q_ values indicate quantification cycle and are also known as the threshold cycle (C_t_). The 75th and 25th percentiles are shown at the top and bottom of each box, respectively. The black line within the box depicts the median. The upper whisker caps represent Q3 + 1.5 × IQR (where Q3 is the third quartile and IQR is the inter-quartile range, or distance between the first and third quartiles) and the lower whisker caps represent Q1 − 1.5× IQR (where Q1 is the first quartile). The black dots indicate outliers.

The mean C_q_ values of all tested samples calculated to determine the expression levels of the 15 CRGs are illustrated in Figure 1. C_q_ value had a negative correlation with gene expression level. In other words, higher gene expression levels are associated with lower C_q_ values and vice versa. The C_q_ values of all CRGs ranged from 18.49 to 32.67. For each CRG, the mean and median C_q_ values were relatively close. Among all the CRGs, *RPS23* demonstrated the highest expression level, with C_q_ = 20.18 ± 0.76, while *PPP1R11* had the lowest expression level, with C_q_ = 30.59 ± 0.89.

### 3.5. Evaluation of Expression Stability for CRGs

In this study, four algorithms: geNorm, NormFinder, BestKeeper and the comparative C_T_ method were used to evaluate CRGs for stability ranking. The stability rankings obtained from the four algorithms were different. Thus, RefFinder was employed to obtain a total score that was used to rank the stability of the 15 CRGs (Table 2).

The M-value was calculated via geNorm analysis to identify gene expression stability. Then, the M-value was used to rank the stability of expression for the 15 CRGs, and the M-value was negatively correlated with the stability of gene expression. According to the geNorm method, the results show that *RPS15* and *DBNDD2* were the most stable CRGs with the lowest M-value of 0.48, while *RPL13A* was the least stable gene with the highest M-value of 0.74 in the yak stomach throughout the growth cycle.

The NormFinder algorithm was used to calculate the stability value (SV) to identify the ranking of the CRGs, with the most stable gene showing the lowest SV. For the yak stomach throughout the growth cycle, the most stable gene was *RPS15* with the lowest SV of 0.35, and *RPL13A* was the most unstable gene with the highest SV of 0.62.

The BestKeeper and the comparative C_T_ method regard standard deviation (SD) as one of the criteria to evaluate the stability of gene expression.. The lower the SD value, the more stable the gene expression. Based on BestKeeper analysis, *RPS15* was the most stable gene, whereas *YWHAZ* was the least stable gene with the highest SD value. By contrast, according to the comparative C_T_ method, *MRPL39* had the highest stability, and the *YWHAZ* was the most unstable gene.

Based on the results obtained using these four algorithms, RefFinder was used for comprehensive ranking. As a result, the comprehensive rankings according to stability from the highest to the lowest are *RPS15* > *MRPL39* > *RPS23* > *DDX54* > *DBNDD2* > *GAPDH* > *TBP* > *RPL13A* > *MRPS15* > *PPP1R11* > *ACTB* > *HMBS* > *RPS9* > *UXT* > *YWHAZ*.

### 3.6. Optimal Number of Reference Genes

**Figure 2:**
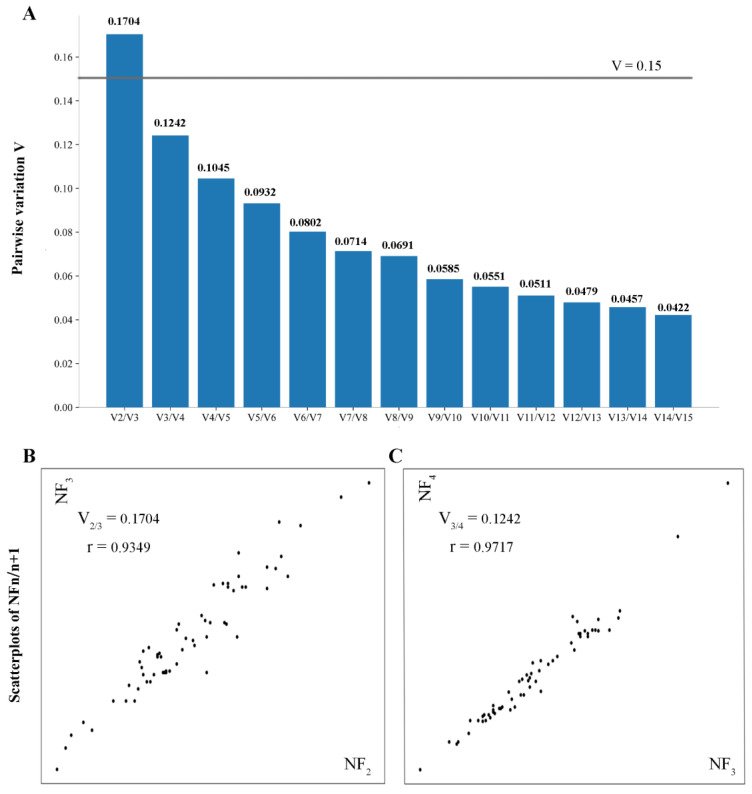
The best number of reference genes for RT-qPCR. (**A**) Pairwise variation (Vn/n + 1) analyses of 15 CRGs. The *y*-axis indicates Vn/Vn + 1 between the calculation of the NFn using the most stable reference genes and the NFn + 1 using an addition of the next most stable reference genes. The gray line depicts the cut-off value (V = 0.15). (**B**,**C**) NF_n/n+1_ scatterplots before and after the addition of (n + 1) reference gene (*x*-axis and *y*-axis). The NF_n_ was calculated using the geometric mean of the RQ data of n CRGs. r: Spearman rank correlation coefficient.

The pairwise variation values (V) were calculated using geNorm software, which is a valid tool to identify the optimal number of reference genes for RT-qPCR. Vandesompele et al. [16] proposed taking 0.15 as a cut-off value below which the inclusion of additional reference genes is not necessary. Thus, according to the cut-off value (V = 0.15), the results indicate that the combination of three genes was the optimal number for normalization of RT-qPCR data in the yak stomach throughout the growth cycle (Figure 2A). Furthermore, low pairwise variation values correspond to a high correlation coefficient [16]. Clearly, there is no need to include an additional gene when using the three most stable reference genes for calculating the NF (Figure 2C). In contrast, it is essential to have more than an additional gene when using the two most stable reference genes for calculation of NF (Figure 2B). Thus, we recommend the combination of the three most stable genes (*RPS15*, *MRPL39*, and *RPS23*) to normalize RT-qPCR data in the yak stomach throughout the growth cycle.

### 3.7. Validation of the Combination of CRGs

**Figure 3:**
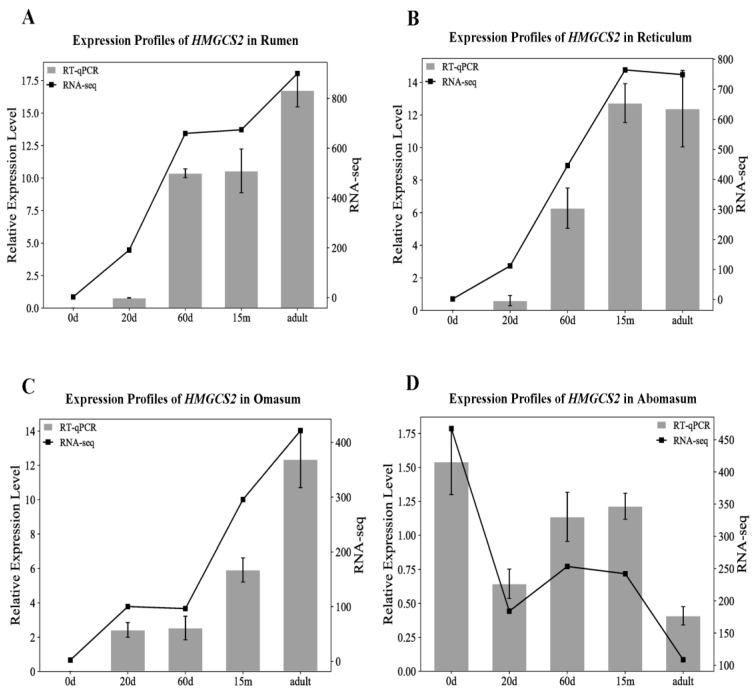
Expression profiles of *HMGCS2* in (**A**) rumen, (**B**) reticulum, (**C**) omasum and (**D**) abomasum at five developmental stages (0 d, 20 d, 60 d, 15 m and adult). Relative expression level: the RT-qPCR data of *HMGCS2* were normalized using *RPS15*, *MRPL39* and *RPS23*. RNA-seq: the arithmetic mean values of FPKM from samples in triplicate at each developmental stage in transcriptome sequencing results.

To verify the effect of the combination of *RPS15*, *MRPL39* and *RPS23* for the normalization of RT-qPCR data, the expression of *HMGCS2* was quantified via RT-qPCR in yak stomach at 5 stages (0 d, 20 d, 60 d, 15 m and adult). Moreover, the expression patterns of *HMGCS2* in yak stomach at 5 stages were also identified using the FPKM of RNA-seq results. The results show a correspondence between the RT-qPCR and RNA-seq, indicating the RT-qPCR data of *HMGCS2* using the *RPS15*, *MRPL39* and *RPS23* for normalization were reliable (Figure 3).

**Figure 4:**
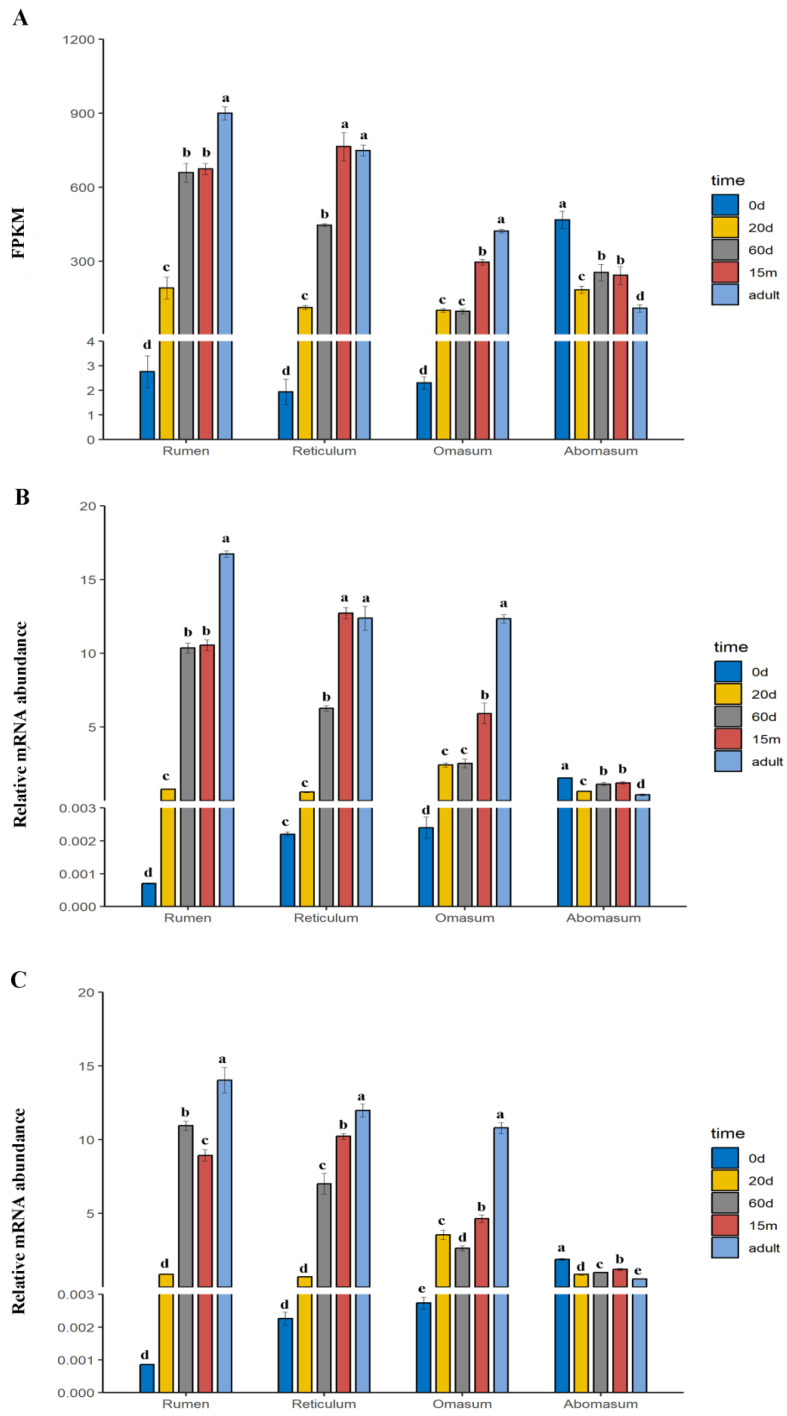
The expression levels of *HMGCS2* (**A**) based on FPKM, (**B**) normalized using the 3 most stable CRGs (*RPS15*, *MRPL39* and *RPS23*), and (**C**) normalized using the 3 least stable CRGs (*RPS9*, *UXT* and *YWHAZ*). The values are means ± SE. Each histogram is divided into four groups (rumen, reticulum, omasum and abomasum). Within each group, the developmental stages with different superscripts indicate significant differences (*p* < 0.05) in *HMGCS2* expression between them. (For example, d is significantly lower than a, b and c).

To further validate the selection of CRGs, the three most stable CRGs (*RPS15*, *MRPL39*, and *RPS23*) and the three least stable CRGs (*RPS9*, *UXT* and *YWHAZ*) were used to normalize the expression of *HMGCS2*. As shown in Figure 4A,B, the expression patterns of *HMGCS2* in the rumen, reticulum, omasum and abomasum at five stages (0 d, 20 d, 60 d, 15 m and adult) were similarly obtained using FPKM based on RNA-seq results and the combination of three most stable CRGs (*RPS15*, *MRPL39*, and *RPS23*) for normalization. Furthermore, the expression of *HMGCS2* in the rumen, reticulum and omasum were the lowest at 0 d and the highest at adulthood, while the opposite was true in the abomasum. However, compared with the expression of *HMGCS2* based on RNA-seq results (Figure 4A), normalization of *HMGCS2* expression using the three least stable CRGs (*RPS9*, *UXT* and *YWHAZ*) demonstrated significant differences (Figure 4C). Hence, it is essential to select suitable reference gene combinations to normalize the expression of target genes.

## 4. Discussion

Gene expression analysis via RT-qPCR is a dependable and extensively used method to reveal the molecular mechanism of digestion and absorption of nutrients in the stomach. The use of reference genes is the most credible strategy for taking into account the initial concentration of RNA, sample loss during experimentation, the efficiency of cDNA synthesis, and so on [28]. However, the selection of inappropriate reference genes also affects the authenticity of RT-qPCR data [29]. Therefore, selecting suitable reference genes is essential to obtain meaningful RT-qPCR results. Until now, strategies for identifying reference genes from the transcriptome have been widely used. Reference genes selected from the transcriptome increase the reproducibility and sensitivity of results, give a stronger correlation between protein expression levels, and have better detection and coverage [30]. Although RNA-seq screening has many merits in predicting reference genes, this strategy is not absolutely trustworthy and needs further validation via RT-qPCR [15,31]. In this study, 15 CRGs were determined via RNA-seq and the previous literature, and further verified using RT-qPCR.

In this study, geNorm, NormFinder, BestKeeper and the comparative C_T_ method were employed to assess the stability of 15 CRGs. Ribosomal protein S15 (*RPS15*) is a component of the 40S ribosomal subunit and functions as a nuclear export factor [32]. Although different algorithms were used for the stability ranking of 15CRGs, *RPS15* had the best stability in all the algorithms except the comparative C_T_ method (geNorm, NormFinder and BestKeeper) (Table 2). Furthermore, *RPS15* was also the most stable gene in the comprehensive ranking of the results of the four algorithms using RefFinder. This is consistent with Bionaz et al. [28] finding that *RPS15* is one of the best reference genes used for the normalization of gene expression data in the bovine mammary gland during the lactation cycle. Tyrosine 3 monooxygenase/tryptophan 5-monooxygenase activation protein zeta (*YWHAZ*), belonging to the 14-3-3 protein family, participates in various cell activities including cell growth, cell cycle, apoptosis, and so on [33,34]. Some studies have demonstrated the consideration of *YWHAZ* as an appropriate reference gene due to its high stability in cattle [35], buffaloes [36] and yak [25]. By comparison, it seems that *YWHAZ* was the least stable gene in our study (Table 2). Even so, the M value of *YWHAZ* (0.73) derived from the geNorm analysis is well below the threshold (M = 1.5) proposed by Vandesompele et al. [16], suggesting that it is also a relatively stable gene in the yak stomach. These results indicate that the stability of reference genes is highly specific and should be evaluated for a given experimental context.

Although *RPS15* had the highest stability in our evaluation, we still do not recommend using it alone as the reference gene for the normalization of RT-qPCR data in the yak stomach. Many studies show that using a single gene as the reference gene should be avoided [16,18,28]. It has been reported that using a single reference gene results in significant bias [37]. To date, no specific theory prescribes a certain number of reference genes to be used. Use of geNorm can provide the optimal number of reference genes needed to eliminate the majority of technical variation [11]. Accuracy and practicality are trade-offs when determining the optimal number of reference genes. It is an unnecessary waste of resources to use more reference genes if the inclusion of additional genes has no significant effect on NF [16]. In our study, there was no significant change between the NF calculated with the three most stable CRGs and that calculated with the four most stable CRGs, indicating that it was superfluous to add a gene for normalization (Figure 2C). In addition, the digestive tract of the yak has three developmental stages: pre-rumination (0–20 days), transition from pre-rumination to rumination (20–60 days), and rumination (after 60 days). The diets of yaks are different at different developmental stages. Therefore, we evaluated the stability of these genes in yak stomach tissue over five developmental stages. Our results support the use of these reference genes in the normalization of RT-qPCR data under different dietary conditions. As a result, we recommend using the combination of three most stable genes (*RPS15*, *MRPL39*, and *RPS23*) to calculate the NF for normalization of RT-qPCR data in the yak stomach throughout the growth cycle.

The expression profiles of *HMGCS2* were quantified via RT-qPCR in the yak stomach at five developmental stages, and its expression levels were normalized by the selected combination of reference genes. *HMGCS2* is the key rate-limiting enzyme in the ketogenic pathway and induces the biosynthesis of HMG-CoA, which is the central metabolite of rumen epithelial cells [38,39]. The ketogenic capacity of ruminal epithelium in ruminants increases with age, and newborn ruminants have no ketogenic capacity [40]. Thus, we hypothesized that the expression level of *HMGCS2* in the rumen should increase in terms of age, as well as those in the reticulum and omasum because they serve analogous functions to the rumen. In this paper, the expression level of *HMGCS2* did increase with age, as determined using RNA-seq and RT-qPCR in the rumen, reticulum and omasum (Figure 3A–C). For newborn ruminants, the rumen was not fully developed and ingested colostrum is instead digested in the abomasum [8]. Consequently, the expression level of *HMGCS2* in the abomasum should be highest at birth and lowest in adulthood (Figure 3D).

In addition, although target genes with significant expression changes can be identified using less stable reference genes, target genes with imperceptible expression changes can only be detected using the best reference genes [20,37]. Our results confirm that significant changes in the expression of *HMGCS2* between birth and adulthood could be identified using either the three most stable CRGs (*RPS15*, *MRPL39*, and *RPS23*) or the three least stable CRGs (*RPS9*, *UXT* and *YWHAZ*). However, when the change in *HMGCS2* expression is slight, errors may occur when using *RPS9*, *UXT* and *YWHAZ* for normalization. For example, in the rumen, there were no significant differences in *HMGCS2* expression levels between 60 days and 15 months either based on the results of RNA-seq or using *RPS15*, *MRPL39*, and *RPS23* for normalization, whereas its expression levels normalized using *RPS9*, *UXT* and *YWHAZ* had significant differences (*p* < 0.05) between 60 days and 15 months (Figure 4). These results imply that using suitable reference genes is essential for accurate normalization of target gene expression.

## 5. Conclusions

In this study, 15 CRGs were selected using transcriptome sequencing results and the previous literature, and their expression stability was evaluated using five algorithms. Therefore, we recommend the combination of the three most stable genes *RPS15*, *MRPL39*, and *RPS23* as reference genes for the normalization of RT-qPCR data in the yak stomach throughout the growth cycle.

## Figures and Tables

**Table 1:** Candidate reference genes and primer pairs characteristics.

Gene	Accession No.	Primer Sequence (5′-3′) ^1^	Size (bp) ^2^	E (%) ^3^	R^2^
* GAPDH *	XM_014482068.1	F: TGGGTGTGAACCACGAGAAG R: CGTGGACGGTGGTCATAAGT	141	95	0.9970
* ACTB *	XM_005887322.2	F: GAGCTACGAGCTTCCTGACG R: CGCAGGATTCCATGCCCAG	104	99	0.9961
* UXT *	XM_005899362.2	F: TGAGCGACTCCAGGAAGCTA R: CCAAGGGCCACATAGATCCG	114	97	0.9955
* DBNDD2 *	XM_014477527.1	F: TTCTTGCCTTGTGAAGACCCTC R: AGGACAAGGAGGAAGTACGAGAC	124	106	0.9996
* RPS9 *	XM_014483477.1	F: CTGAAGCTGATCGGCGAGTA R: GGGTCTTTCTCATCCAGCGT	119	101	0.9940
* DDX54 *	XM_005904734.2	F: CCTTGCACGAAAATCCCGAC R: AGCCCATTTCAAAGAGCCTGT	135	97	0.9937
* HMBS *	XM_005897125.2	F: TTGGATCTGGTGGGTGTGTT R: CTCCAGTCAGGTACAGTTGCC	148	100	0.9949
* RPS15 *	XM_005890466.2	F: GCGGAAGTGGAACAGAAGAA R: GCATCAGTTGCTCATAGGACAT	100	91	0.9979
* MRPS15 *	XM_014477429.1	F: CTCAAGTCCTGGAGGTCTCAT R: CTGGTAGTCCTTCAGCAGCAT	115	99	0.9967
* RPS23 *	XM_005903762.2	F: TGTGCTGGAAAAAGTAGGAGTT R: AGCAACCATCATTGGGTACAA	122	109	0.9999
* PPP1R11 *	XM_014483599.1	F: AGTGGGTTTGGGAGAATCGC R: GTTAGGCTCCGGTTCTCAGAC	143	92	0.9965
* MRPL39 *	XM_005898618.2	F: AGAGCCCCAGAAGTTCCAGT R: AGAACGCAGGTTCTCTTTTGTTG	102	92	0.9948
* TBP *	XM_005908678.2	F: AAGATAACCCACAGAGCCGAG R: GCTCCTCCAGAATAGACAGACTGTT	286	97	0.9958
* YWHAZ *	XM_005887010.2	F: CCTACTCCGGACACAGAACAT R: CAGGCTGCCATGTCATCATATC	101	99	0.9985
* RPL13A *	XM_005904989.2	F: GGTTCCTTCTTTCCCAGGCA R: CAACCTTGCGGCCCAGAA	130	107	0.9984

^1^ F: forward primer, R: reverse primer. ^2^ size: amplicon size. ^3^ E (%): amplification efficiency (%) = (10^(−1/slope)^ − 1) × 100%.

**Table 2:** Stability of CRGs in yak stomach throughout the growth cycle.

CRGs	GeNorm		NormFinder		BestKeeper		Delta Ct		Comprehensive Ranking	
	R-Based		R-Based		Excel Plug-in		Excel Plug-in		RefFinder	
	Rank	Value	Rank	Value	Rank	Value	Rank	Value	Rank	Value
* RPS15 *	1	0.48	1	0.35	1	0.51	2	0.78	1	1.41
* MRPL39 *	3	0.53	2	0.38	8	0.67	1	0.77	2	2.51
* RPS23 *	4	0.56	8	0.55	4	0.55	7	0.85	3	3.74
* DDX54 *	6	0.61	5	0.49	6	0.58	3	0.81	4	3.83
* DBNDD2 *	1	0.48	3	0.44	3	0.54	6	0.85	5	5.05
* GAPDH *	13	0.72	11	0.58	5	0.57	9	0.87	6	5.90
* TBP *	7	0.63	6	0.50	12	0.70	4	0.83	7	6.31
* RPL13A *	15	0.74	15	0.62	2	0.52	11	0.88	8	6.42
* MRPS15 *	10	0.69	10	0.57	9	0.68	5	0.85	9	6.89
* PPP1R11 *	5	0.59	4	0.48	11	0.70	8	0.86	10	8.85
* ACTB *	12	0.71	12	0.59	7	0.63	10	0.87	11	9.37
* HMBS *	9	0.67	9	0.56	10	0.69	12	0.91	12	11.92
* RPS9 *	8	0.65	7	0.53	14	0.76	13	0.93	13	12.98
* UXT *	11	0.70	13	0.61	13	0.73	14	0.93	14	13.49
* YWHAZ *	14	0.73	14	0.62	15	0.80	15	0.93	15	15.00

## Data Availability

Data sharing is not applicable to this article.

## Supplementary Materials

The following supporting information can be downloaded at: https://www.mdpi.com/article/10.3390/ani13050925/s1, Figure S1: Agarose gel electrophoresis for all tested RNA samples; Figure S2: Agarose gel electrophoresis for 15 candidate reference genes; Figure S3: Melting curves of 15 candidate reference gen; Table S1: The purity and concentration of RNA samples used in this study; Table S2: The stably expressed genes identified in the yak stomach via RNA-sequencing; Table S3: Sequence alignment results in NCBI for 15 candidate reference genes.

## References

[1] Guo X., Long R., Kreuzer M., Ding L., Shang Z., Zhang Y., Yang Y., Cui G. (2014). Importance of functional ingredients in yak milk-derived food on health of Tibetan nomads living under high-altitude stress: A review. Crit. Rev. Food Sci. Nutr..

[2] Qiu Q., Zhang G., Ma T., Qian W., Wang J., Ye Z., Cao C., Hu Q., Kim J., Larkin D.M. (2012). The yak genome and adaptation to life at high altitude. Nat. Genet..

[3] Nozière P., Ortigues-Marty I., Loncke C., Sauvant D. (2010). Carbohydrate quantitative digestion and absorption in ruminants: From feed starch and fibre to nutrients available for tissues. Anim. Int. J. Anim. Biosci..

[4] Ma L., Xu S., Liu H., Xu T., Hu L., Zhao N., Han X., Zhang X. (2019). Yak rumen microbial diversity at different forage growth stages of an alpine meadow on the Qinghai-Tibet Plateau. PeerJ.

[5] Guo L., Yao J., Cao Y. (2021). Regulation of pancreatic exocrine in ruminants and the related mechanism: The signal transduction and more. Anim. Nutr. (Zhongguo Xu Mu Shou Yi Xue Hui).

[6] Swanson K.C. (2019). Small Intestinal Anatomy, Physiology, and Digestion in Ruminants. Reference Module in Food Science.

[7] Teixeira A.F., Kühnel W., Vives P., Wedel T. (2009). Functional morphology of unguiculiform papillae of the reticular groove in the ruminant stomach. Ann. Anat. = Anat. Anz. Off. Organ Anat. Ges..

[8] Meale S.J., Chaucheyras-Durand F., Berends H., Steele M.A. (2017). From pre- to postweaning: Transformation of the young calf’s gastrointestinal tract. J. Dairy Sci..

[9] Cholewińska P., Czyż K., Nowakowski P., Wyrostek A. (2020). The microbiome of the digestive system of ruminants—A review. Anim. Health Res. Rev..

[10] Wagner E.M. (2013). Monitoring gene expression: Quantitative real-time rt-PCR. Methods Mol. Biol..

[11] Derveaux S., Vandesompele J., Hellemans J. (2010). How to do successful gene expression analysis using real-time PCR. Methods.

[12] Bustin S.A., Benes V., Garson J.A., Hellemans J., Huggett J., Kubista M., Mueller R., Nolan T., Pfaffl M.W., Shipley G.L. (2009). The MIQE guidelines: Minimum information for publication of quantitative real-time PCR experiments. Clin. Chem..

[13] Bai W.L., Yin R.H., Zhao S.J., Jiang W.Q., Yin R.L., Ma Z.J., Wang Z.Y., Zhu Y.B., Luo G.B., Yang R.J. (2014). Technical note: Selection of suitable reference genes for studying gene expression in milk somatic cell of yak (Bos grunniens) during the lactation cycle. J. Dairy Sci..

[14] Hruz T., Wyss M., Docquier M., Pfaffl M.W., Masanetz S., Borghi L., Verbrugghe P., Kalaydjieva L., Bleuler S., Laule O. (2011). RefGenes: Identification of reliable and condition specific reference genes for RT-qPCR data normalization. BMC Genom..

[15] Zhang J., Deng C., Li J., Zhao Y. (2020). Transcriptome-based selection and validation of optimal house-keeping genes for skin research in goats (Capra hircus). BMC Genom..

[16] Vandesompele J., De Preter K., Pattyn F., Poppe B., Van Roy N., De Paepe A., Speleman F. (2002). Accurate normalization of real-time quantitative RT-PCR data by geometric averaging of multiple internal control genes. Genome Biol..

[17] Andersen C.L., Jensen J.L., Ørntoft T.F. (2004). Normalization of real-time quantitative reverse transcription-PCR data: A model-based variance estimation approach to identify genes suited for normalization, applied to bladder and colon cancer data sets. Cancer Res..

[18] Pfaffl M.W., Tichopad A., Prgomet C., Neuvians T.P. (2004). Determination of stable housekeeping genes, differentially regulated target genes and sample integrity: BestKeeper--Excel-based tool using pair-wise correlations. Biotechnol. Lett..

[19] Silver N., Best S., Jiang J., Thein S.L. (2006). Selection of housekeeping genes for gene expression studies in human reticulocytes using real-time PCR. BMC Mol. Biol..

[20] Li Y., Han J., Wu J., Li D., Yang X., Huang A., Bu G., Meng F., Kong F., Cao X. (2020). Transcriptome-based evaluation and validation of suitable housekeeping gene for quantification real-time PCR under specific experiment condition in teleost fishes. Fish Shellfish. Immunol..

[21] Mezera M.A., Li W., Edwards A.J., Koch D.J., Beard A.D., Wiltbank M.C. (2020). Identification of stable genes in the corpus luteum of lactating Holstein cows in pregnancy and luteolysis: Implications for selection of reverse-transcription quantitative PCR reference genes. J. Dairy Sci..

[22] Zhao J., Wang C., Zhang L., Lei A., Wang L., Niu L., Zhan S., Guo J., Cao J., Li L. (2021). Genome-Wide Identification of Reference Genes for Reverse-Transcription Quantitative PCR in Goat Rumen. Animals.

[23] Pfaffl M.W. (2001). A new mathematical model for relative quantification in real-time RT-PCR. Nucleic Acids Res..

[24] Jiang M., Lee J.N., Bionaz M., Deng X.Y., Wang Y. (2016). Evaluation of Suitable Internal Control Genes for RT-qPCR in Yak Mammary Tissue during the Lactation Cycle. PLoS ONE.

[25] Wu X., Zhou X., Ding X., Chu M., Liang C., Pei J., Xiong L., Bao P., Guo X., Yan P. (2019). The Selection of Reference Genes for Quantitative Real-Time PCR in the Ashidan Yak Mammary Gland during Lactation and Dry Period. Animals.

[26] Wu X., Zhou X., Ding X., Chu M., Liang C., Pei J., Xiong L., Bao P., Guo X. (2020). Reference gene selection and myosin heavy chain (MyHC) isoform expression in muscle tissues of domestic yak (Bos grunniens). PLoS ONE.

[27] Die J.V., Baldwin R.L., Rowland L.J., Li R., Oh S., Li C., Connor E.E., Ranilla M.-J. (2017). Selection of internal reference genes for normalization of reverse transcription quantitative polymerase chain reaction (RT-qPCR) analysis in the rumen epithelium. PLoS ONE.

[28] Bionaz M., Loor J.J. (2007). Identification of reference genes for quantitative real-time PCR in the bovine mammary gland during the lactation cycle. Physiol. Genom..

[29] Ma S., Niu H., Liu C., Zhang J., Hou C., Wang D. (2013). Expression stabilities of candidate reference genes for RT-qPCR under different stress conditions in soybean. PLoS ONE.

[30] Fu X., Fu N., Guo S., Yan Z., Xu Y., Hu H., Menzel C., Chen W., Li Y., Zeng R. (2009). Estimating accuracy of RNA-Seq and microarrays with proteomics. BMC Genom..

[31] Gao D., Kong F., Sun P., Bi G., Mao Y. (2018). Transcriptome-wide identification of optimal reference genes for expression analysis of Pyropia yezoensis responses to abiotic stress. BMC Genom..

[32] Léger-Silvestre I., Milkereit P., Ferreira-Cerca S., Saveanu C., Rousselle J.C., Choesmel V., Guinefoleau C., Gas N., Gleizes P.-E. (2004). The ribosomal protein Rps15p is required for nuclear exit of the 40S subunit precursors in yeast. EMBO J..

[33] Fu H., Subramanian R.R., Masters S.C. (2000). 14-3-3 proteins: Structure, function, and regulation. Annu. Rev. Pharmacol. Toxicol..

[34] Gan Y., Ye F., He X.X. (2020). The role of YWHAZ in cancer: A maze of opportunities and challenges. J. Cancer.

[35] De Ketelaere A., Goossens K., Peelman L., Burvenich C. (2006). Technical note: Validation of internal control genes for gene expression analysis in bovine polymorphonuclear leukocytes. J. Dairy Sci..

[36] Macabelli C.H., Ferreira R.M., Gimenes L.U., de Carvalho N.A.T., Soares J.G., Ayres H., Ferraz M.L., Watanabe Y.F., Watanabe O.Y., Sangalli J.R. (2014). Reference gene selection for gene expression analysis of oocytes collected from dairy cattle and buffaloes during winter and summer. PLoS ONE.

[37] Kozera B., Rapacz M. (2013). Reference genes in real-time PCR. J. Appl. Genet..

[38] Xiang R., Oddy V.H., Archibald A.L., Vercoe P.E., Dalrymple B.P. (2016). Epithelial, metabolic and innate immunity transcriptomic signatures differentiating the rumen from other sheep and mammalian gastrointestinal tract tissues. PeerJ.

[39] Pan X., Cai Y., Li Z., Chen X., Heller R., Wang N., Wang Y., Zhao C., Wang Y., Xu H. (2021). Modes of genetic adaptations underlying functional innovations in the rumen. Sci. China Life Sci..

[40] Lane M.A., Baldwin IV R.L., Jesse B.W. (2002). Developmental changes in ketogenic enzyme gene expression during sheep rumen development. J. Anim. Sci..

