# Supplementary figures and images for: Transcriptome-Based Evaluation of Optimal Reference Genes for Quantitative Real-Time PCR in Yak Stomach throughout the Growth Cycle
Source: Animals (Basel). 2023 Mar 3;13(5):925. doi: 10.3390/ani13050925 (PMC10000025; doi:10.3390/ani13050925)

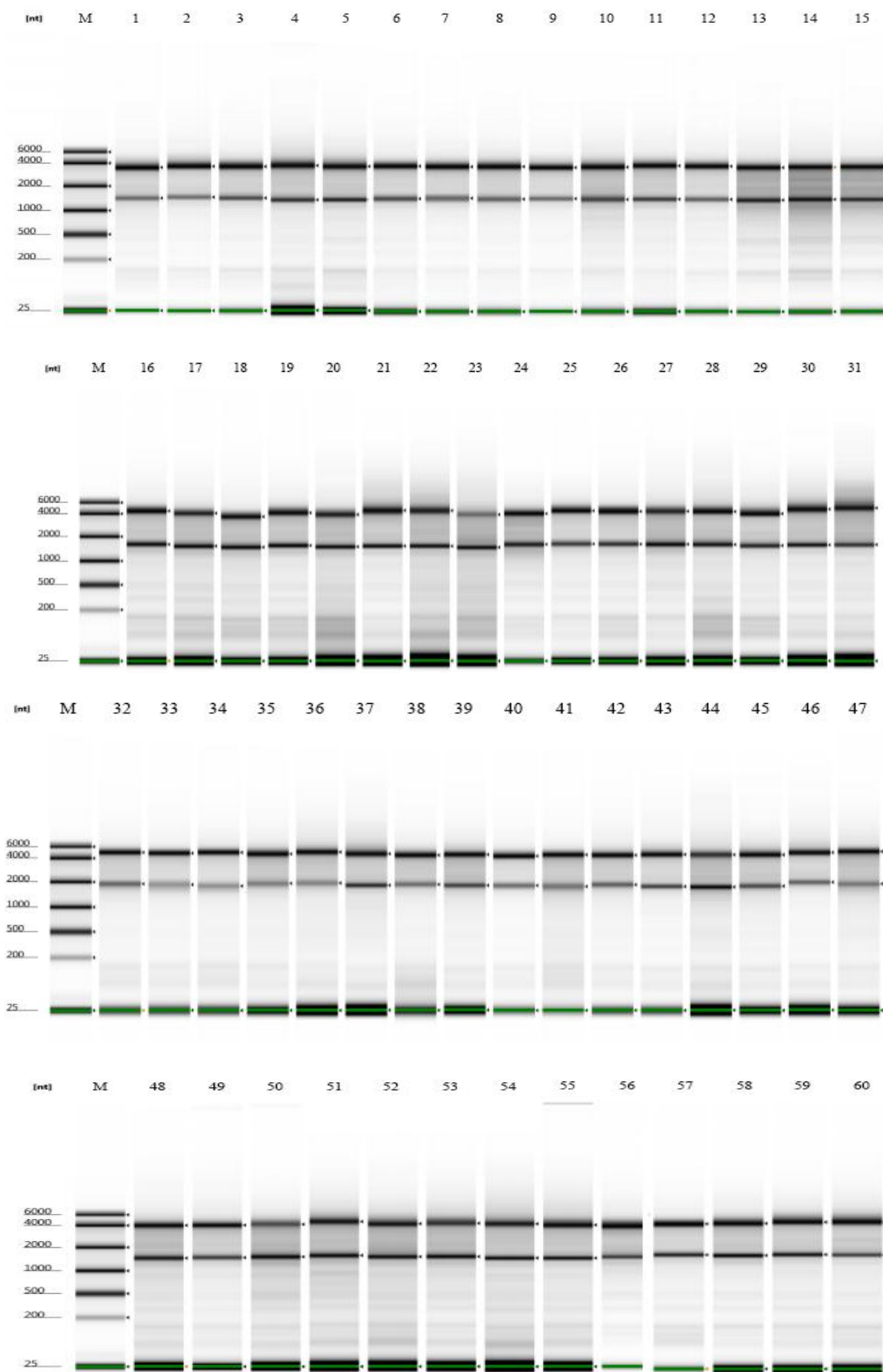

**Figure S1.** Agarose gel electrophoresis for all tested RNA samples

Supplement: Supplementary file 1 [file animals-13-00925-s001.zip › Figure S1.pdf]

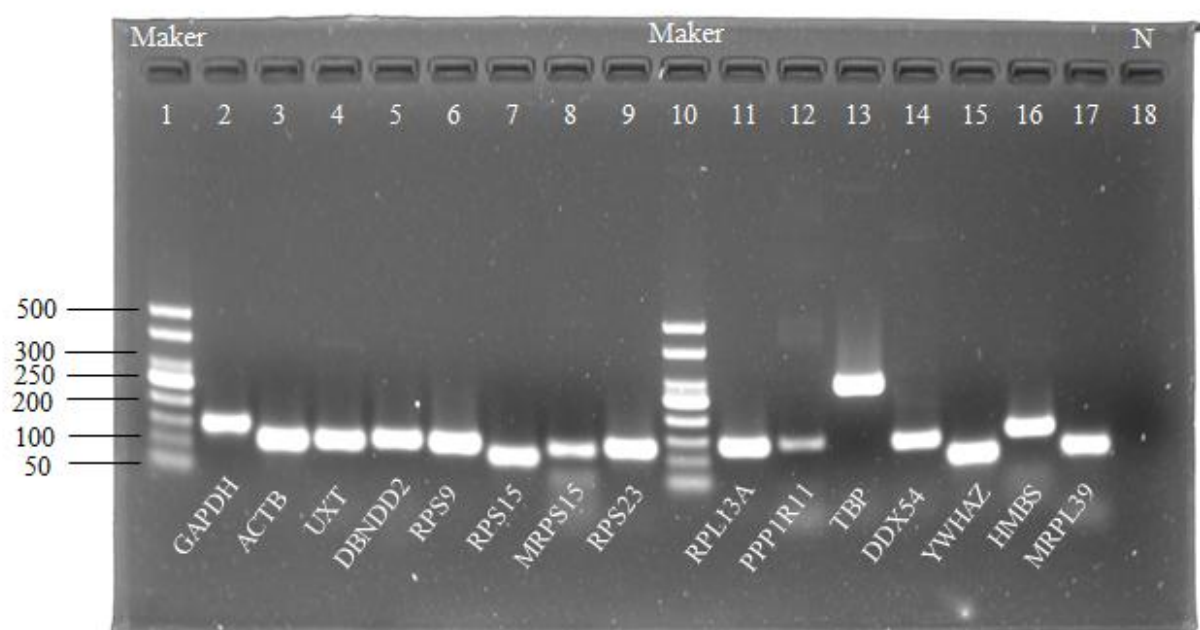

**Figure S2.** Agarose gel electrophoresis for 15 candidate reference genes

Supplement: Supplementary file 1 [file animals-13-00925-s001.zip › Figure S2.pdf]

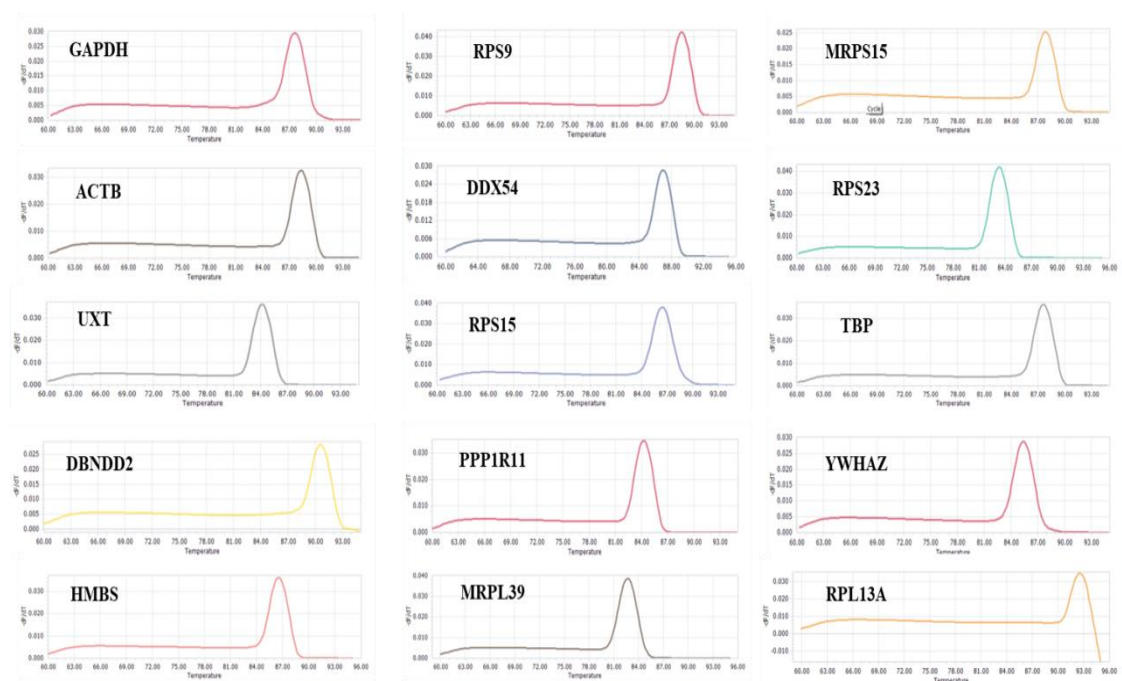

**Figure S3.** Melting curves of 15 candidate reference genes

Supplement: Supplementary file 1 [file animals-13-00925-s001.zip › Figure S3.pdf]
